# The Role of decision-analytic modelling in German health technology assessments

**DOI:** 10.1186/s13561-014-0039-x

**Published:** 2015-02-19

**Authors:** Alexander Kuhlmann, Marina Treskova, Sebastian Braun, J-Matthias Graf von der Schulenburg

**Affiliations:** 1 Center for Health Economics Research Hannover (CHERH), Leibniz Universität Hannover, Hannover, Germany; 2 Xcenda GmbH- Health Economic Research & Consulting, Hannover, Germany

**Keywords:** Health technology assessment, Health economic evaluation, Health economic modelling, Cost-effectiveness, Cost-utility, Decision analysis

## Abstract

**Background:**

Decision-analytic modelling (DAM) has become a widespread method in health technology assessments (HTA), but the extent to which modelling is used differs among international HTA institutions. In Germany, the use of DAM is optional within HTAs of the German Institute of Medical Documentation and Information (DIMDI). Our study examines the use of DAM in DIMDI HTA reports and its effect on the quality of information provided for health policies.

**Methods:**

A review of all DIMDI HTA reports (from 1998 to September 2012) incorporating an economic assessment was performed. All included reports were divided into two groups: HTAs with DAM and HTAs without DAM. In both groups, reports were categorized according to the quality of information provided for healthcare decision making.

**Results:**

Of the sample of 107 DIMDI HTA reports, 17 (15.9%) used DAM for economic assessment. In the group without DAM, conclusions were limited by the quality of economic information in 51.1% of the reports, whereas we did not find limited conclusions in the group with DAM. Furthermore, 24 reports without DAM (26.7%) stated that using DAM would likely improve the quality of information of the economic assessment.

**Conclusion:**

The use of DAM techniques can improve the quality of HTAs in Germany. When, after a systematic review of existing literature within a HTA, it is clear that DAM is likely to positively affect the quality of the economic assessment DAM should be used.

**Electronic supplementary material:**

The online version of this article (doi:10.1186/s13561-014-0039-x) contains supplementary material, which is available to authorized users.

## Background

In the process of health technology assessment, decision-analytic modelling serves as an assessment approach for economic evaluation. Performing economic evaluation in the HTA has become a standard requirement of healthcare systems in many countries (e.g. the UK, Canada, Australia), and DAM has been accepted as a valid analytical approach. The method is applied to synthesize existing evidence on the costs and effectiveness of healthcare options and to determine an optimal strategy among them. In recent years, the use of DAM for HTA has significantly increased [1,2], and several studies providing good practice guidelines for the use of DAM in HTA have been conducted [2]. In particular, guidelines issued by HTA institutes in the UK and Canada provide detailed descriptions of the required elements of HTAs and the appropriate methods for decision modelling.

In Germany, HTA was introduced in the 1990s. In 1995, the German Federal Ministry of Health assembled a research group and assigned it to review, assess and prepare the implementation of data collection and to evaluate medical procedures and technologies in Germany [3]. HTA was formally approved in Germany with the healthcare reform in 2000. The German Agency for Health Technology Assessment (DAHTA) was established within the German Institute of Medical Documentation and Information (DIMDI). It was commissioned to implement and operate a database, an information system and a scientific working program on HTA [4,5]. The HTAs published by DIMDI aim to primarily inform health policy and not to provide recommendations for the benefits catalogue of the Statutory Health Insurance (SHI) [5]. These HTA reports include medical, economic, ethical, social and juridical aspects [6]. Following the SHI Modernization Act in 2004, HTA gained increasing importance in Germany. The Institute for Quality and Efficiency in Health Care (IQWiG) was established as an independent scientific body to perform technology assessments on behalf of the Federal Joint Committee (G-BA; a supreme decision making body of the self-governing healthcare system in Germany) or the Federal Ministry of Health. The technology assessments serve to inform the decision making by the G-BA [5], and the reports by IQWiG were limited to medical technology assessments. Since 2007, with the German Act on reinforcing SHI competition, IQWiG may also be commissioned to perform cost-benefit assessments.

The guidelines of both DIMDI and IQWiG indicate that DAM may be necessary for economic assessment of a technology [6,7]; however, the incorporation of a model is not a requirement for developing an HTA for publication by DIMDI, and DAM has become an optional tool in practice. IQWiG sees modelling as essential for economic assessment and requires it in the absence of comprehensive economic data [7]. Thus, IQWiG has released detailed information on the methods applied in modelling [8]. The DIMDI guidelines, which are summarized in its handbook, do not provide specific methodological recommendations for the development of DAM.

Considering the growing importance of performing systematic assessments of health technologies in Germany, it is desirable to continue working on the development of HTA methodologies, which may improve the quality of HTA reports. One important direction may be to enhance the application of decision models in German HTAs. Therefore, in our study, we review the use of DAM in German HTAs and analyse the effects of the use of DAM on the quality of information provided for healthcare decision making. We also study decision models applied to German settings and consolidate the main characteristics of the models developed by the German HTAs. Because IQWiG did not provide economic assessments by the time of our analysis, we based our work on the HTA reports published by DIMDI.

## Methods

### Search strategy and exclusion criteria

Using the DAHTA database, we identified and extracted all DIMDI HTA reports conducted during the period from 1998 to September 2012. HTAs that did not undertake economic assessments were excluded from the analysis. The resulting sample of HTAs was divided into two groups: HTAs that performed a systematic literature review and HTAs that developed a new decision-analytic model (designed for the characteristics of the German healthcare system) in addition to the literature review for the economic assessment.

### Assessing the informativeness of HTA reports for decision making

In order to analyse the informativeness of HTAs for decision making, we developed an HTA classification based on the quality of information provided in each HTA report. We reviewed all included HTAs, focusing on the following sections: the summary, the conclusion and the answers provided to the research questions. Additionally, we checked for consistency between these sections. We defined three aggregate types that described the levels of informativeness of the HTA report.

**‘Conclusion’**: The HTA provides a clear conclusion regarding the medical effectiveness or cost-effectiveness of the health technology (technologies) under assessment. Uncertainty is low and further research is unlikely to affect the given conclusion.

**‘Limited conclusion’**: Authors on an HTA formulate a general suggestion regarding the medical effectiveness/cost-effectiveness of the health technology (technologies) under assessment, but the conclusion is limited because of the limitations of the reviewed evidence. Major limitations of the study are explained through either the low quality of the reviewed studies or the difficulties of applying the existing evidence to the German health care system. The latter generally occurs when conducting an economic assessment because of the differences in the healthcare structures and/or resource prices. In addition, the uncertainty is significant, and further research is likely to have a considerable effect on the results and may change the provided inferences.

**‘No conclusion’**: Authors of an HTA cannot provide an assessment of medical effectiveness and cost-effectiveness of the health technology (technologies) because of the lack of scientific evidence in the reviewed literature.

### Assessing the impact of decision-analytic modelling on the informativeness of HTA reports

The medical effectiveness of interventions is a key input parameter in decision-analytic models. The evidence of medical effectiveness affects the quality of information provided in health economic evaluations. Low-quality medical evidence can be a barrier for conducting economic analysis. Consequently, the existing medical evidence has to be taken into account when assessing the impact of decision-analytic modelling on the informativeness of HTA reports. Therefore, we reviewed the medical part and the economic part of each HTA separately. Using the three types of informativeness, six categories (CAT I-VI) were formed to classify the HTA reports. Table 1 shortly sketches these categories. The first row and column of the table provide the level of information related to the medical and economic assessments, respectively. Combinations between the type of the medical part and the type the economic part constitute the six categories shown in the intersection cells of the table.

**Table 1:** **Categorization of HTA conclusions based on the ‘quality level’ of information for decision making**

		**Economic assessment**		
		**Conclusion**	**Limited conclusion**	**No conclusion**
**Medical assessment**	Conclusion	I	II	III
	Limited conclusion	not applicable	IV	V
	No conclusion	not applicable	not applicable	VI

In order to evaluate the impact of using DAM on the quality of information given in the economic portions of the HTAs, we compared medical and economic assessments of each report in the sample and analysed the difference between their levels of informativeness. HTA reports in CAT VI were excluded from the analysis, since the reports in this category provide insufficient medical evidence for progressing to economic evaluation. For each of the groups, ‘HTA with a model’ and ‘HTA without a model’, we determined the percentage of HTAs in which the economic assessment provides significantly lower quality of information than the medical assessment (CATs II, III and V). We compared “with-” and “without a model” groups based on these percentages to reduce potential bias, in case both groups are not comparable with respect to the reported level of information in the medical assessment.

The review and the classification of the reports were undertaken by two researchers independently, and any distinctions were discussed and clarified. The HTA reports with new model development were further analysed with respect to the applied modelling methods. The aim of the further analyses was to characterize and compare the techniques used that focused on the selected key components of modelling: the economic evaluation type, the model type, the time horizon, the perspective, the primary medical outcome, the discount factor and the type of sensitivity analyses. These components were extracted according to the individual descriptions provided in the HTA reports.

## Results

### Sample size

**Figure 1:**
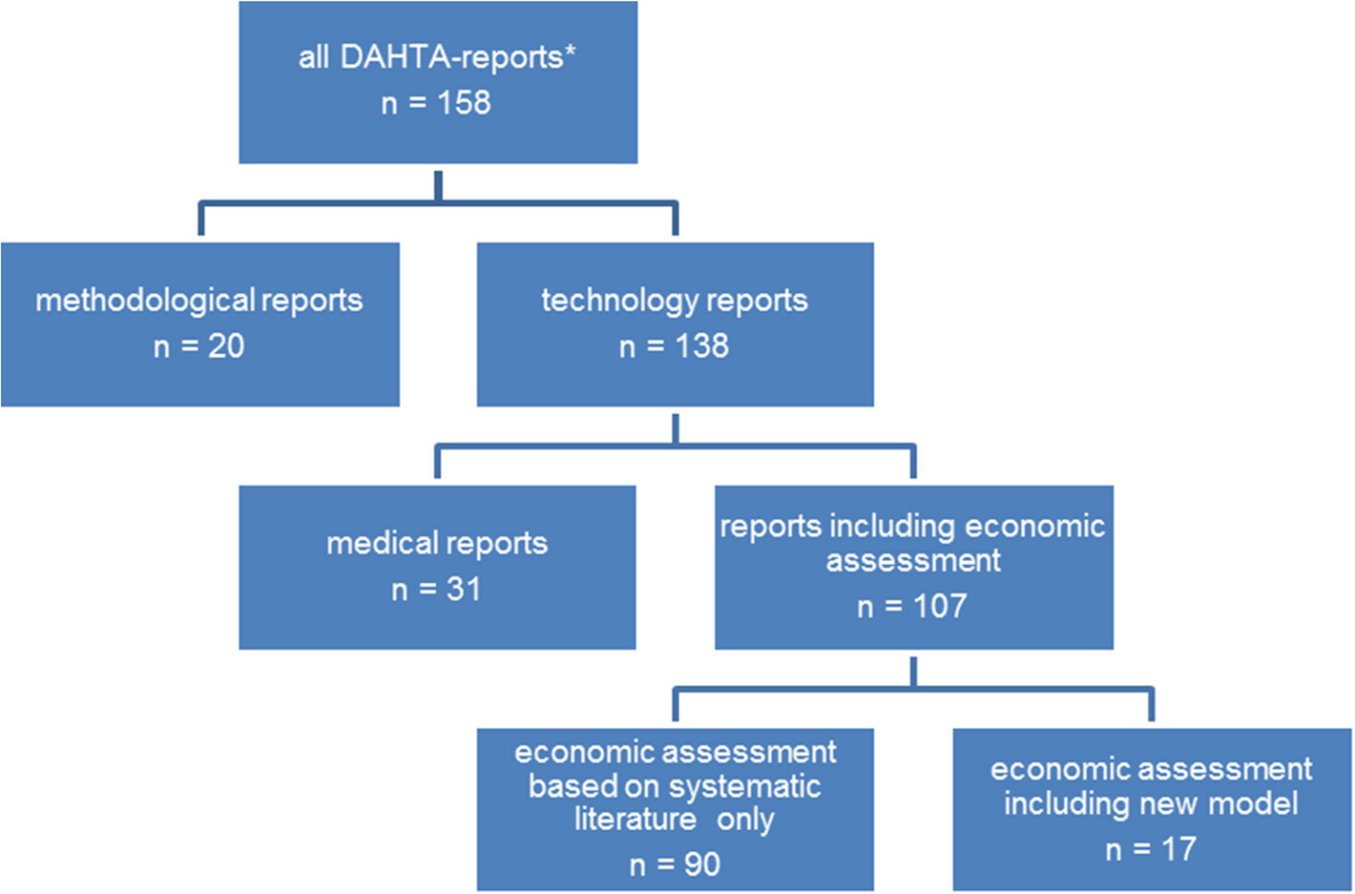
**Selection process for report inclusion.** *As of September 2012.

In the period from 1998 to September 2012, 158 DIMDI–HTA reports were conducted, published and indexed in the DAHTA database. Of these, 20 methodological reports were excluded during the screening process. Another 31 reports did not meet the inclusion criterion of an economic assessment of the health technology. The resulting sample of 107 HTA reports was divided into reports that include the development of a new model for the German healthcare system and those that only performed a systematic literature review for economic assessment. In total, 17 HTA reports (approximately 16%) developed such a model, whereas the other 90 reports did not. Figure 1 summarizes the selection procedure (A list of all identified reports is presented in the Additional file 1: Table S2 to Table S5).

### Informativeness of DAHTA reports

**Figure 2:**
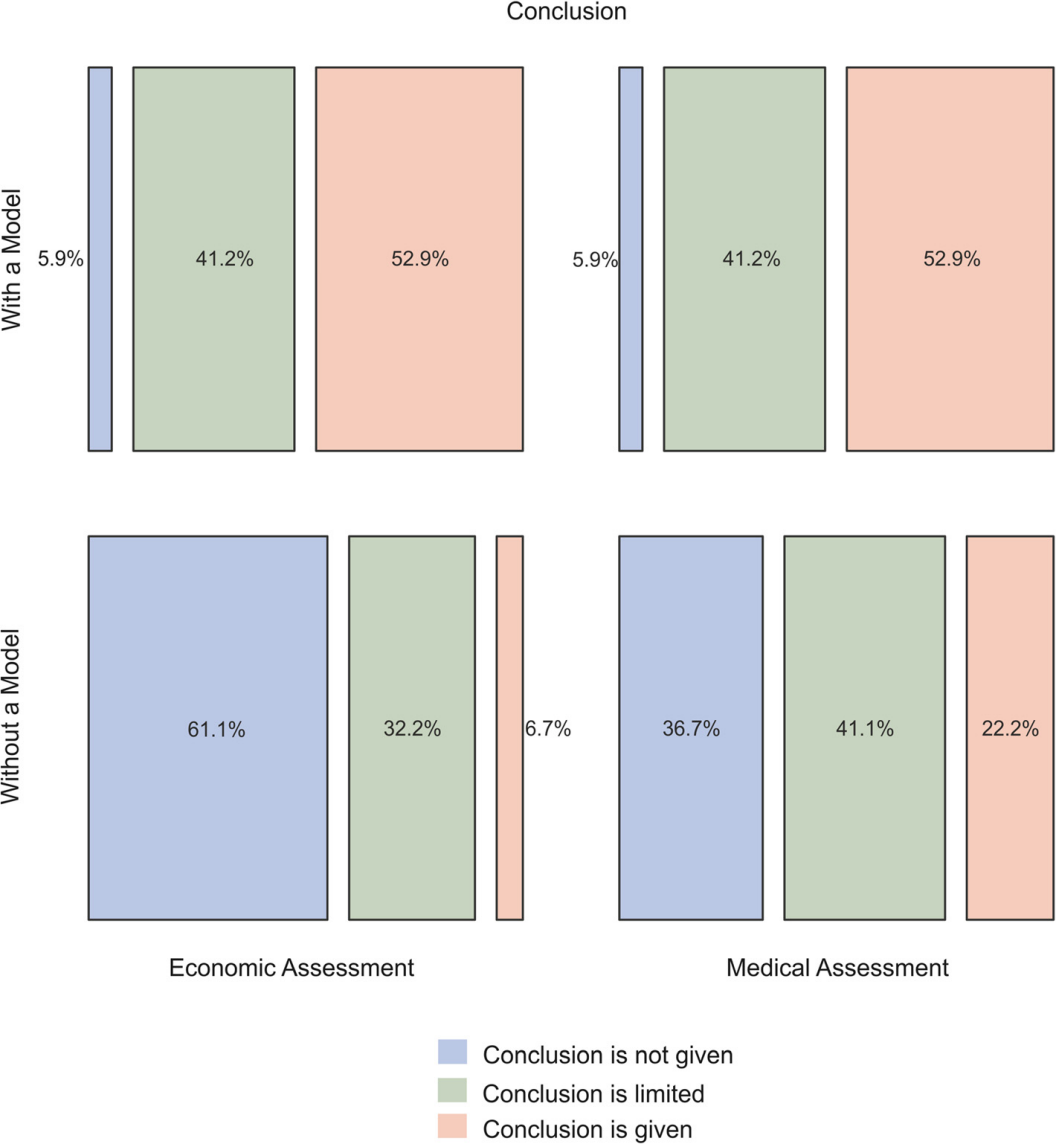
**Results of the division of HTA reports into the types.**

The medical and economic parts of the 107 HTAs in our sample are grouped into the three aggregate types. Of our sample, 29 reports (27.1%) state a clear conclusion regarding the medical effectiveness of a technology in the assessment, and 15 reports (14%) provide a conclusion regarding cost-effectiveness in the economic section. Another 44 reports (41.1%) state a general suggestion on medical effectiveness. The conclusion on cost-effectiveness is significantly limited in 36 reports (33.6%). In 34 reports (31.8%), it is not possible to conduct a medical assessment because of the lack of scientific evidence in the reviewed literature. An economic conclusion could not be drawn in 56 reports (52.3%), either because of the lack of economic evidence or because the results of the international studies are not applicable to the German setting. Figure 2 shows the results of the division of the reports into the types for the groups of ‘HTAs with a model’ and ‘HTAs without a model’. In the sample of HTAs with a model, a higher percentage of reports provide information for decision making in the medical assessment compared to the sample of HTAs without a model (conclusion is given in 52.9% vs. 22.2%; conclusion is limited in 41.2% vs. 41.1%). Of the reports that applied DAM, 94.1% provide either a clear conclusion (32.2%) or a limited conclusion (61.1%) on the cost-effectiveness of the technology/technologies under assessment compared with 38.9% in the group without a model (conclusion: 6.7%; limited conclusion: 32.2%).

Overall, the proportion of HTA reports that provide information on the assessed health technology for decision making (CAT I–V) is 68.2% (73 out of 107 reports), of which 15 reports draw a clear conclusion on both the medical and economic assessments (CAT I). Another 14 reports either draw a clear conclusion on effectiveness but provide only a general suggestion on cost-effectiveness (CAT II; seven reports), or they are not able to provide a conclusion on cost-effectiveness based on the reviewed evidence (CAT III; seven reports). Of the 44 reports that provide a general suggestion on medical effectiveness, 29 reports also provide a general suggestion on cost-effectiveness (CAT IV) and 15 are not able to assess the cost-effectiveness because of a lack of evidence (CAT V). In addition, 31.8% (34 of 107 reports) of the reports are grouped in CAT VI, because they cannot draw a conclusion on medical effectiveness or cost-effectiveness.

Among the 90 HTAs without a new model 57 (63.3%) give information for decision making (CAT I–V). The majority of these (51 reports) provide only a general opinion on the medical effectiveness or cost-effectiveness of the health technology (technologies) under assessment (CAT II–V); thus, further research is likely to have an important effect and may change the conclusion. The number of the reports in CATs II, III, IV and V are 7, 7, 22 and 15, respectively.

The HTA reports with a developed model provide information for decision making (CAT I–V) in 16 out of the 17 cases (94.1%). Of these, nine HTAs draw a clear conclusion and provide high-quality information in both the medical and economic assessment (CAT I), and seven HTAs provide a general suggestion on both effectiveness and cost-effectiveness (CAT IV).

### Impact of decision-analytic modelling on informativeness of DAHTA reports

**Figure 3:**
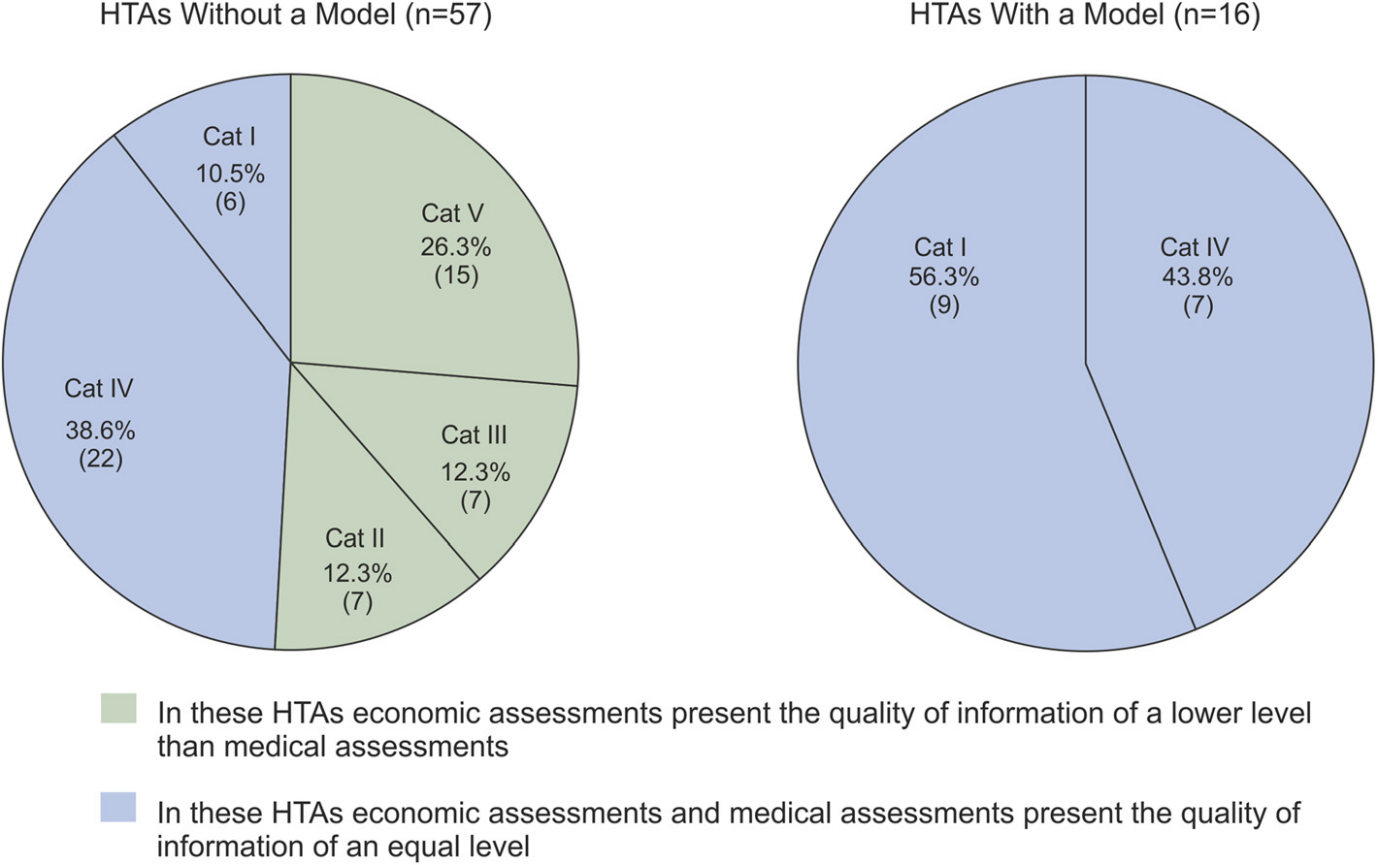
**Impact of decision-analytic modelling on the conclusion of HTA reports.**

Of the HTA reports that did not develop a decision-analytic model for the German healthcare system, 51.1% provide significantly less information in the economic assessment compared with the medical assessment. Of the 20 reports that provide a conclusion in the medical assessment, 7 provide suggestions in the economic assessment and another 7 cannot draw a conclusion. Of the 37 reports with a limited conclusion on effectiveness in the medical assessment, 15 reports provide no information on cost-effectiveness in the economic assessment. In the group of HTAs that apply DAM techniques, no report provides significantly less information in the economic assessment compared with the medical assessment. Figure 3 illustrates the results for both groups.

Additionally, we reviewed the proportion of HTAs that reported a requirement for further economic research. The majority of these reports (86 of 107) conclude that additional economic evidence is required in the literature. Of the 90 reports that conduct only an economic systematic literature research, 26.7% (24 reports) state that the development of a model is likely to improve the quality of information of the economic assessment. Six of the HTAs that used DAM provide recommendations to update the models as soon as new medical evidence is available.

Economic evaluations for the German settings are retrieved in 36 of 107 HTA reports; however, none of these evaluations significantly affected the level of information in the economic assessments, mainly because of either the low quality of the evaluation or the use of outdated economic or medical data.

### Characteristics of decision-analytic models

Seventeen of the HTA reports in our sample developed new decision-analytic models for the German settings. Here, we provide a review of these and focus on the selected key components of health economic modelling. Table 1 in the supplements summarizes the results. One model was not completed because of the lack of medical evidence of important input parameters, and this model is excluded from the following review.

#### Type of economic evaluation

Overall, 10 cost-effectiveness analyses and 2 cost-utility analyses were conducted within the 16 HTAs. Three HTA reports include examination of costs, and one report includes both a cost-effectiveness analysis and a cost-utility analysis.

#### Model type

The applied DAM techniques are identified in 63% (10 of 16) of the HTAs. In six HTAs, economic evaluations are based on Markov models. Three HTA reports apply decision trees. One report uses combinations of a decision tree and the Markov model. Another report presents a Markov chain Monte Carlo simulation in which, in contrast with common cohort modelling, virtual patients are simulated on an individual level with the implication of a stochastic process (i.e. a micro-simulation). The remaining six HTAs calculate results on the basis of simple calculations.

#### Discount factor

Overall, 11 of the 16 models apply a discount rate in the economic evaluations. Of these, six models use a 3% annual discount rate and five models use a 5% discount rate for both health effects and costs. In one model, it is unnecessary to discount because the economic evaluation is performed for a short time horizon (1 year). One model omits discounting of the costs and benefits, albeit it performs the economic evaluation for a time horizon of 3 years. The other three models report no discount rate.

#### Perspective

Of the 16 reviewed models, four state a social perspective. Three models describe the perspective as a narrowed social perspective. The perspective of two models is that of the German statutory health insurance. One model uses the scope of a healthcare provider and includes additional costs in its evaluation. Five models state no perspective; however, the outcomes of the models probably reflect the perspective of the statutory health insurance. One model uses the perspective of the German healthcare system.

#### Primary medical outcome

Three models use life years to value health outcomes and two models use QALYs. One model bases the economic evaluation on both life years and QALYs. The rest of the models calculate health outcomes specific to the character of the disease or the technology under assessment.

#### Type of sensitivity analysis

All 16 models conduct sensitivity analyses. All models apply a one-way sensitivity analysis; in addition, three reports also perform a multi-way analysis and two perform a probabilistic sensitivity analysis.

## Discussion

Following the objective of this work, we searched for evidence that developing a new decision-analytical model improves the quality of information of HTAs for decision making in the German healthcare sector.

Therefore, we reviewed all HTAs published from DIMDI that included economic assessments and classified them according to the quality level of the information provided for decision making. The results of this study suggest that HTAs perform better when they build a new decision model for economic evaluation. Particularly, the review showed that all HTAs with developed models were capable of providing economic evidence for decision making with the quality of the information at least equivalent to that provided by the medical portion. In contrast, over 50% of HTA reports without model gave a lower level of information in the economic assessments than in the medical assessment. Moreover, approximately 80% of the reviewed HTAs concluded that there is a need for further economic research, and 27% (24 reports) of the HTAs without a model stated that the development of a decision-analytic model might improve the quality of the information of the economic assessment. These findings indicate that using DAM in DAHTA reports is not related to the need for additional economic information.

In our analysis, we also found differences between the models in their quality and complexity. The review of HTAs with models indicated that cost-effectiveness analysis with Markov models was the preferred type of economic evaluation. Although the majority of the selected HTAs with models incorporated the key elements of modelling, some differences in the applied methods were observed. These differences occurred in valuing medical outcomes, the stated perspectives and the applied annual discount rate. Not all the applied methods were up-to-date. For example, for addressing uncertainty, a one-way sensitivity analysis but no probabilistic sensitivity analysis was mostly conducted.

Current shortcomings of the HTA reports and the differences between the applied methods might complicate decision making processes and might decrease the role of HTAs as sources of information in healthcare. Elaboration of official standards and recommendations on the use of decision-analytic models in HTA might solve the discrepancies in the applied methods. Imposing a requirement of justifying and clarifying the necessity for modelling seems to be useful. Thus, requiring DAM is necessary when, after conducting a systematic literature review, it is justified that a model would improve the results of assessment in terms of informing decision making.

The current description of the HTA methods by DIMDI lacks guidance on both, methods for conducting decision modelling and for assessment of cost-effectiveness (e.g. ICER vs. the Efficiency Frontier of the IQWiG [7]). Since these aspects are interconnected, they are both essential for the production of consistent results among HTAs. For instance, if the assessment of cost-effectiveness allows for comparisons between health outcomes, a generic measure such as QALY should be applied in modelling. When developing a guide on decision modelling in HTA, both the modelling methods and the assessment of outcomes must be considered. Additionally, it is desirable to consider the requirements and needs of the users of HTA reports. For instance, requests by decision makers may determine the applied perspective (e.g. societal or sickness funds).

Some limitations of this study should be considered when contemplating the results. First, because of the diversity and complexity of the HTAs conclusions provided, the types and categories of our classifications are broadly defined. A more precise grouping might change the results of the classifications, but it would unlikely affect the overall conclusion of our study. Second, the classifications were performed based on the concluding statements provided by the authors of the HTAs; therefore, we did not conduct an assessment of the evidence reviewed in the reports. Among the HTA researchers, distinctions in valuing the existing evidence may exist. These differences might in turn bias our work.

Despite the limitations, this study provides new information on conducting HTA in Germany regarding the use of DAM. It also indicates the lack of economic research in the German HTAs as well as the need for increased and improved economic evaluations conducted for HTAs.

## Conclusion

Our review shows that it is necessary to improve economic evaluations for HTAs produced in Germany. The results of the analysis suggest that the use of modelling improves the quality of economic assessment and thereby the overall performance of an HTA, however, the number of HTAs that conduct modelling is small. In order to enhance the quality of HTAs in Germany, it is desirable to develop a procedure for incorporating decision-analytic models in the economic assessments of reports. As long as the application of modelling is not necessary for every HTA study, it seems reasonable to develop a model on request after a systematic literature review clarifies that DAM is likely to have a positive impact on the economic assessment quality. In order to guarantee good modelling quality and consistency of the applied methods, designing and expanding the good practices guide for the use of DAM for DIMDI–HTAs is required.

## Additional file

Additional file 1:
**Technology reports with economic assessment based on systematic literature review.**
