# Supplementary material for: The Role of decision-analytic modelling in German health technology assessments
Source: Health Econ Rev. 2015 Feb 19;5:7. doi: 10.1186/s13561-014-0039-x (PMC4385016; doi:10.1186/s13561-014-0039-x)
Supplement: Additional file 1: — Technology reports with economic assessment based on systematic literature review. [file 13561_2014_39_MOESM1_ESM.docx]

**Supplement**

**List of acronyms and abbreviations**

| DAHTA | Deutsche Agentur für Health Technology Assessment (Eng: German Agency for Health Technology Assessment) |
| --- | --- |
| DAM | Decision-Analytic Modelling |
| DIMDI | Deutschen Institut für medizinische Dokumentation und Information (Eng:German Institute of Medical Documentation and Information) |
| G-BA | Gemeinsame Bundesausschuss (Eng:Federal Joint Committee) |
| HTA | Health Technology Assessment |
| ICER | Incremental Cost-Effectiveness Ratio |
| IQWiG | Institut für Qualität und Wirtschaftlichkeit im Gesundheitswesen (Eng:Institute for Quality and Efficiency in Health Care) |
| SHI | Statutory Health Insurance |

**Table 1: Summary of basic modelling methods in Health Technology Assessments of the German Institute of Medical Documentation and Information (DIMDI)**

| **Author** | **Year** | **Indication** | **Intervention type** | **Type of economic evaluation** | **Model type** | **Time horizon** | **Perspective** | **Primary clinical outcome** | **Discount factor** | **Type of sensitivity analyses** |
| --- | --- | --- | --- | --- | --- | --- | --- | --- | --- | --- |
| **Aidelsburger et al.*** | 2003 | Osteoporosis | Screening | Cost-effectiveness | Hybrid (decision tree + markov model) | Life time | ns | ns | ns | ns |
| **Siebert et al.** | 2003 | Cervical carcinoma | Screening | Cost-effectiveness | Markov model | Life time | Social | Life years | 3% | One-way, two-way |
| **Siebert et al.** | 2003 | Hepatitis C | Treatment | Cost-effectiveness, cost-utility | Markov model | Life time | Social | Life years, QALY | 3% | One-way, multi-way |
| **Corzillius et al.** | 2003 | HIV | Diagnostics | Cost-effectiveness | Markov model | Life time | Health care system | Life years | 5% | One-way, multi-way, probabilistic |
| **Dauben et al.** | 2004 | Hearing disorder | Screening | Cost-effectiveness | Markov model | 10 (16) years | ns | time with correct diagnosis | 3% | One-way |
| **Frank et al.** | 2004 | Cervical carcinoma | Screening | Cost-effectiveness | Decision tree | 40 years | ns | Correct diagnosed cases | ns | One-way |
| **Gorenoi et al.** | 2005 | Coronary heart disease | Treatment | Cost-effectiveness | ns | 1 year, life time | ns | Avoided revasculations | ns | One-way |
| **Zahn et al.** | 2006 | Obstretrics | Treatment | Cost-analysis | Decision tree | 2 weeks, 50 years | Care provider + additional costs | Not relevant | 3% | One-way |
| **Schnell-Inderst et al.** | 2006 | Hearing Disorder | Screening | Cost-effectiveness | Markov Model | 10 years | ns | Time with correct diagnosis | 3% | One-way |
| **Gorenoi et al.** | 2006 | Appendicitits | Treatment | Cost-analysis | ns | ns | ns | Not relevant | ns | One-way |
| **Siebert et al.** | 2008 | Coronary heart disease | Treatment | Cost-utility | Hybrid (decision tree + markov model) | Life time | Social | QALY | 5% | One-way |
| **Gorenoi et al.** | 2008 | Peripheral arterial disease | Treatment | Cost-effectiveness | ns | 1 year | Narrowed social | Prevented restenosis, revascularization | Not relevant | One-way |
| **Gorenoi et al.** | 2008 | Coronary heart disease | Treatment | Cost-analysis | ns | 1 year, 3 years | Narrowed social | Number of deaths, heart attacks** | None | One way |
| **Schmieder et al.** | 2010 | Brain tumor (meningioma) | Treatment | Cost-utility | Markov chain monte carlo simulation | Life time | Statutory health insurance | QALY | 5% | One-way, probabilistic |
| **Sroczynski et al.** | 2010 | Cervical carcinoma / HPV | Screening | Cost-effectiveness | Markov model | Life time | Statutory health insurance | Life years | 3% | One-way, multi-way |
| **Gorenoi et al.** | 2011 | Stable angina pectoris | Treatment | Cost-effectiveness | ns | 5 years | Narrowed social | Prevented angina pectoris episodes | 5%*** | One-way |
| **Gorenoi et al.** | 2012 | Coronary heart disease | Diagnostics | Cost-effectiveness | ns | 10 years | Social | Correct diagnosis | 5% | One-way |

* Due to a lack of medical evidence, only the structure of the model was reported.

** Cost-effectiveness not calculated.

*** Only in sensitivity analysis.

**Table 2: Technology reports with economic assessment based on systematic literature review (n=90); included**

| **Document Number** | **Authors** | **Year** | **Titel** |
| --- | --- | --- | --- |
| DAHTA023 | Perleth M; Kochs G | 1999 | Stenting versus Ballondilatation bei koronarer Herzkrankheit. Systematische Übersichten zur medizinischen Effektivität und zur Kosten-Effektivität |
| DAHTA016 | Bitzer EM; Greiner W | 2000 | Hochdosis-Chemotherapie mit autologer Stammzelltransplantation zur Therapie des metastasierenden Mammakarzinoms |
| DAHTA019 | Siebert U; Mühlberger N; Behrend C; Wasem J | 2001 | PSA-Screening beim Prostatakarzinom - systematischer gesundheitsökonomischer Review |
| DAHTA020 | Müller A; Stratmann-Schöne D; Klose T; Leidl R | 2001 | Ökonomische Evaluationen der Positronen-Emissions-Tomographie |
| DAHTA021 | Perleth M; Leyen U von der; Schmitt H; Dintsios CM; Felder S; Schwartz FW; Teske S | 2003 | Das Schlaf-Apnoe-Syndrom - systematische Übersichten zur Diagnostik, Therapie und Kosten-Effektivität |
| DAHTA028 | Kulp W; Garrido Velasco M; Greiner W; Schulenburg JM Graf von der | 2003 | Die Verwendung des Excimer Lasers in der refraktiven Augenchirurgie |
| DAHTA032 | Kulp W; Greiner W; Schulenburg JM Graf von der | 2003 | Bewertung der Möglichkeiten und Verfahren zur Aufbereitung medizinischer Einwegprodukte |
| DAHTA033 | Olbrich A; Felder S | 2003 | Knochen- und Knochenersatzmaterialien zur parodontalen Regeneration oder zum Knochenaufbau für Implantate |
| DAHTA064 | Felder S; Meyer FP | 2003 | Glycoprotein IIb/IIIa-Rezeptorantagonisten in der Therapie akuter koronarer Syndrome - ein gesundheitsökonomischer HTA-Bericht |
| DAHTA066 | Siebert U; Aidelsburger P; Peeters J; Regar E; Mühlberger N; Klauss V; Rieber J; Corzillius M; Wasem J | 2003 | Wertigkeit des Einsatzes der intravaskulären Ultraschallbildgebung (IVUS) im Rahmen von diagnostischen und therapeutischen Herzkatheteruntersuchungen - ein gesundheitsökonomischer HTA-Bericht |
| DAHTA076 | Gerhardus A; Jalilvand N; Heintze C; Krauth C | 2003 | Ein Vergleich verschiedener chirurgischer Verfahren zur elektiven Leistenhernienoperation bei Erwachsenen - ein Health Technology Assessment |
| DAHTA077 | Gorenoi V; Siebert U; Perleth M; Brundobler M; Dintsios CM; Klauss V; Rieber J; Wasem J; Leidl R | 2003 | Stenting versus Ballondilatation bei koronarer Herzkrankheit |
| DAHTA075 | Kulp W; Corzillius M; Greiner W; Pientka L; Siebert U; Schulenburg JM Graf von der; Wasem J | 2005 | Wertigkeit von Tumor-Nekrose-Faktor-alpha-Antagonisten in der Behandlung der Rheumatoiden Arthritis |
| DAHTA084 | Vauth C; Englert H; Fischer T; Kulp W; Greiner W; Willich SN; Stroever B; Schulenburg JM Graf von der | 2005 | Sonographische Diagnostik beim akuten Abdomen bei Kindern und Erwachsenen |
| DAHTA108 | Lühmann D; Burkhardt-Hammer T; Borowski C; Raspe H | 2005 | Minimal-invasive Verfahren zur Behandlung des Bandscheibenvorfalls |
| DAHTA109 | Habl C; Bodenwinkler A; Stürzlinger H | 2005 | Wurzelbehandlung an Molaren |
| DAHTA111 | Gorenoi V; Kulp W; Greiner W; Schulenburg JM Graf von der | 2005 | Thrombozytenaggregationshemmer zur Primär- und Sekundärprävention des ischämischen Schlaganfalls |
| DAHTA114 | Antony K; Pichlbauer E; Stürzlinger H | 2005 | Medizinische und ökonomische Effektivität der Pneumokokkenimpfung für Säuglinge und Kleinkinder |
| DAHTA115 | Frank W; Konta B | 2005 | Bluthochdruckleitlinien und ihre Auswirkungen auf das Gesundheitssystem |
| DAHTA116 | Braun S; Behrens T; Kulp W; Eberle A; Greiner W; Ahrens W; Schulenburg JM Graf von der | 2005 | Neuraminidasehemmer in der Therapie und Postexpositionsprohylaxe der Influenza |
| DAHTA119 | Hessel F; Grabein K; Schnell-Inderst P; Siebert U; Caspary W; Wasem J | 2005 | Extrakorporale artifizielle Leberunterstützungssysteme bei akutem Leberversagen oder einer akuten Dekompensation eines chronischen Leberleidens |
| DAHTA123 | Frank W; Konta B | 2005 | Kognitives Training bei Demenzen und andere Störungen mit kognitiven Defiziten |
| DAHTA128 | Eberhardt S; Heinemann A; Kulp W; Greiner W; Leffmann C; Leutenegger M; Anders J; Pröfener F; Balmaceda U; Cordes O; Zimmermann U; Schulenburg JM Graf von der | 2005 | Dekubitusprophylaxe und -therapie |
| DAHTA130 | Carvalho Gomes H de; Velasco-Garrido M; Busse R | 2005 | Screening auf urogenitale Chlamydia trachomatis-Infektionen |
| DAHTA118 | Frank W; Konta B; Seiler G | 2006 | Therapie des unspezifischen Tinnitus ohne Ursache |
| DAHTA120 | Eidt D; Roll S; Kulp W; Müller-Nordhorn J; Vauth C; Greiner W; Willich SN; Schulenburg JM Graf von der | 2006 | Bypassmaterialien in der Gefäßchirugie |
| DAHTA121 | Aidelsburger P; Grabein K; Huber A; Hertlein H; Wasem J | 2006 | Die elastisch stabile intramedulläre Nagelung bei instabilen kindlichen Unterarmschaftfrakturen |
| DAHTA124 | Stürzlinger H; Antony K; Pichlbauer E | 2006 | Koronarkalkbestimmung mit CT-Verfahren bei asymptomatischen Risikopatienten |
| DAHTA125 | Heinen-Kammerer T; Wiosna W; Nelles S; Rychlik R | 2006 | Monitoring von Herzfunktionen mit Telemetrie |
| DAHTA129 | Claes C; Kulp W; Greiner W; Schulenburg JM Graf von der; Werfel T | 2006 | Therapie der mittelschweren und der schweren Psoriasis |
| DAHTA132 | Frank W; Konta B; Prusa N; Raymann C | 2006 | Bedeutung der intensivierten Pflege |
| DAHTA133 | Rosian I; Pichlbauer E; Stürzlinger H | 2006 | Einsatz von Statinen in der Primärprävention |
| DAHTA134 | Lühmann D; Burkhardt-Hammer T; Stoll S; Raspe H | 2006 | Prävention rezidivierender Rückenschmerzen- Präventionsmaßnahmen in der Arbeitsplatzumgebung |
| DAHTA135 | Schnell-Inderst P; Kossmann B; Fischereder M; Klauss V; Wasem J | 2006 | Antioxidative Vitamine zur Prävention kardiovaskulärer Erkrankungen nach Nierentransplantation und bei chronischer Niereninsuffizienz |
| DAHTA136 | Walter U; Krauth C; Wienold M; Dreier M; Bantel S; Droste S | 2006 | Verfahren zur Steigerung der Teilnahmerate an Krankheitsfrüherkennungsprogrammen |
| DAHTA140 | Rohde V; Grabein K; Hessel F; Siebert U; Wasem J | 2006 | Orchiektomie versus medikamentöse Therapie mit LH-RH-Analoga zur Behandlung des fortgeschrittenen Prostatakarzinoms |
| DAHTA141 | Frank W; Konta B | 2006 | Bypassoperation am schlagenden Herzen im Vergleich zur Operation mit Unterstützung durch die Herz-Lungen-Maschine |
| DAHTA142 | Werfel T; Claes C; Kulp W; Greiner W; Schulenburg JM Graf von der | 2006 | Therapie der Neurodermitis |
| DAHTA145 | Bockelbrink A; Rasch A; Roll S; Willich SN; Greiner W | 2006 | Welche Auswirkung hat die Kataraktoperation auf das Entstehen oder das Fortschreiten einer altersbedingten Makuladegeneration (AMD)? |
| DAHTA127 | Eberhardt S; Keil T; Kulp W; Greiner W; Willich SN; Schulenburg JM Graf von der | 2007 | Hormone zur Therapie von Beschwerden im Klimakterium und zur Primärprävention von Erkrankungen in der Postmenopause |
| DAHTA138 | Rosian-Schikuta I; Fröschl B; Habl C; Stürzlinger H | 2007 | Die Masern-Mumps-Röteln-Impfung aus gesundheitspolitischer und ökonomischer Sicht |
| DAHTA144 | Antony K; Genser D; Fröschl B | 2007 | Erkennungsgüte und Kosteneffektivität von Screeningverfahren zur Erfassung von primären Offenwinkelglaukomen |
| DAHTA146 | Busch M; Haas S; Weigl M; Wirl C; Horvath I; Stürzlinger H | 2007 | Langzeitsubstitutionsbehandlung Opioidabhängiger |
| DAHTA147 | Schumacher H; Müller-Nordhorn J; Roll S; Willich SN; Greiner W | 2007 | Drotrecogin alfa (aktiviert) bei der Behandlung der schweren Sepsis |
| DAHTA149 | Stürzlinger H; Fröschl B; Genser D | 2007 | Wertigkeit der optischen Kohärenztomographie im Vergleich zur Fluoreszenzangiographie in der Diagnostik der altersbedingten Makuladegeneration (AMD) |
| DAHTA187 | Gorenoi V; Schönermark MP; Hagen A | 2007 | Nutzen und Risiken hormonaler Kontrazeptiva bei Frauen |
| DAHTA189 | Angermayr L; Velasco Garrido M; Busse R | 2007 | Künstliche Ventrikel bei fortgeschrittener Herzinsuffizienz |
| DAHTA195 | Lühmann D; Schramm S; Raspe H | 2007 | Wie ist der derzeitige Stellenwert der Homozysteinbestimmung im Blut als Risikofaktor für die koronare Herzkrankheit (KHK)? |
| DAHTA198 | Nocon M; Mittendorf T; Roll S; Greiner W; Willich SN; Schulenburg JM Graf von der | 2007 | Welchen medizinischen und gesundheitsökonomischen Nutzen hat die Kolposkopie als primäres Screening auf das Zervixkarzinom? |
| DAHTA199 | Mittendorf T; Nocon M; Roll S; Mühlberger N; Sroczynski G; Siebert U; Willich SN; Schulenburg JM Graf von der | 2007 | HPV-DNA-Diagnostik zur Zervixkarzinomfrüherkennung |
| DAHTA206 | Gorenoi V; Schönermark MP; Hagen A | 2007 | Maßnahmen zur Verbesserung der Compliance bzw. Adherence in der Arzneimitteltherapie mit Hinblick auf den Therapieerfolg |
| DAHTA143 | Friedrich M; Müller-Riemenschneider F; Roll S; Kulp W; Vauth C; Greiner W; Willich SN; Schulenburg JM Graf von der | 2008 | Vergleich der laparoskopischen Narbenhernioplastik und der konventionellen Operation mit und ohne Netzeinlage – Effektivität und Kostennutzenrelation |
| DAHTA186 | Fröschl B; Arts D; Leopold C | 2008 | Topische antientzündliche Behandlung der Neurodermitis im Kindesalter |
| DAHTA203 | Bockelbrink A; Stöber Y; Roll S; Vauth C; Willich SN; Greiner W | 2008 | Medizinische und ökonomische Beurteilung der bariatrischen Chirurgie (Adipositaschirurgie) gegenüber konservativen Strategien bei erwachsenen Patienten mit morbider Adipositas |
| DAHTA204 | Konta B; Frank W | 2008 | Die Therapie der Parkinsonerkrankung mit Dopaminagonisten |
| DAHTA205 | Frank W; Pfaller K; Konta B | 2008 | Mundgesundheit nach kieferorthopädischer Behandlung mit festsitzenden Apparaten |
| DAHTA213 | Clar C; Velasco-Garrido M; Gericke C | 2008 | Interferone und Natalizumab in der Behandlung der multiplen Sklerose (MS) |
| DAHTA224 | Antony K; Hiebinger C; Genser D; Windisch F | 2008 | Haltbarkeit von Zahnamalgam im Vergleich zu Kompositkunststoffen |
| DAHTA232 | Müller-Riemenschneider F; Rasch A; Bockelbrink A; Vauth C; Willich SN; Greiner W | 2008 | Wirksamkeit und Wirtschaftlichkeit von verhaltensbezogenen Maßnahmen zur Prävention des Zigarettenrauchens |
| DAHTA215 | Rieckmann N; Schwarzbach C; Nocon M; Roll S; Vauth C; Willich SN; Greiner W | 2009 | Pflegerische Versorgungskonzepte für Personen mit Demenzerkrankungen |
| DAHTA216 | Schnell-Inderst P; Schwarzer R; Göhler A; Grandi N; Grabein K; Stollenwerk B; Klauss V; Wasem J; Siebert U | 2009 | Stellenwert des hochsensitiven C-reaktiven Proteins (hs-CRP) als Marker für Herzinfarktgefährdung |
| DAHTA217 | Stürzlinger H; Genser D; Hiebinger C; Windisch F | 2009 | ffektivität und Effizienz der CT-Koloskopie im Vergleich zur konventionellen Koloskopie in der Dickdarmkrebsdiagnose und -früherkennung |
| DAHTA225 | Müller-Riemenschneider F; Schwarzbach C; Bockelbrink A; Ernst I; Vauth C; Willich SN; Schulenburg JM Graf von der | 2009 | Medizinische und gesundheitsökonomische Bewertung der Radiochirurgie zur Behandlung von Hirnmetastasen |
| DAHTA228 | Stürzlinger H; Hiebinger C; Pertl D; Traurig P | 2009 | Computerized Physician Order Entry – Wirksamkeit und Effizienz elektronischer Arzneimittelverordnung mit Entscheidungsunterstützungssystemen |
| DAHTA234 | Damm O; Nocon M; Roll S; Vauth C; Willich SN; Greiner W | 2009 | Impfung gegen humane Papillomaviren (HPV) zur Prävention HPV 16/18 induzierter Zervixkarzinome und derer Vorstufen |
| DAHTA236 | Brunner-Ziegler S; Fröschl B; Hiebinger C; Wimmer A; Zsifkovits J | 2009 | Effektivität und Kosteneffizienz von Phosphatbindern in der Dialyse |
| DAHTA242 | Fröschl B; Haas S; Wirl C | 2009 | Prävention von Adipositas bei Kindern und Jugendlichen (Verhalten- und Verhältnisprävention) |
| DAHTA248 | Weinmann S; Schwarzbach C; Begemann M; Roll S; Vauth C; Willich SN; Greiner W | 2009 | Verhaltens- und fertigkeitenbasierte Frühinterventionen bei Kindern mit Autismus |
| DAHTA257 | Nocon M; Kuhlmann A; Leodolter A; Roll S; Vauth C; Willich SN; Greiner W | 2009 | Medizinischer und gesundheitsökonomischer Nutzen der Untersuchung auf Helicobacter pylori-Besiedlung mittels 13C-Harnstoff-Atemtest in der Primärdiagnostik im Vergleich zu invasiven und nichtinvasiven diagnostischen Verfahren |
| DAHTA261 | Müller-Riemenschneider F; Damm K; Meinhard C; Bockelbrink A; Vauth C; Willich SN; Greiner W | 2009 | Nichtmedikamentöse Sekundärprävention der koronaren Herzkrankheit (KHK) |
| DAHTA254 | Hagen A; Gorenoi V; Schönermark MP | 2010 | Spezifische Immuntherapie (SIT) zur Behandlung der allergischen Rhinitis |
| DAHTA256 | Tinnemann P; Stöber Y; Roll S; Vauth C; Willich SN; Greiner W | 2010 | Zahnmedizinische Indikationen für standardisierte Verfahren der instrumentellen Funktionsanalyse unter Berücksichtigung gesundheitsökonomischer Gesichtspunkte |
| DAHTA258 | Grimm C; Köberlein J; Wiosna W; Kresimon J; Kiencke P; Rychlik R | 2010 | Diabetesneuentstehung unter antihypertensiver Therapie |
| DAHTA262 | Buchberger B; Follmann M; Freyer D; Huppertz H; Ehm A; Wasem J | 2010 | Bedeutung von Wachstumsfaktoren für die Behandlung von chronischen Wunden am Beispiel des diabetischen Fußulcus |
| DAHTA263 | Korczak D; Schöffmann C | 2010 | Medizinische Wirksamkeit und Kosten-Effektivität von Präventions- und Kontrollmaßnahmen gegen Methicillin-resistente Staphylococcus aureus (MRSA)-Infektionen im Krankenhaus |
| DAHTA267 | Benkert D; Krause KH; Wasem J; Aidelsburger P | 2010 | Medikamentöse Behandlung der ADHS (Aufmerksamkeitsdefizit-/Hyperaktivitätsstörung) im Erwachsenenalter in Deutschland |
| DAHTA268 | Geiseler J; Karg O; Börger S; Becker K; Zimolong A | 2010 | Invasive Heimbeatmung insbesondere bei neuromuskulären Erkrankungen |
| DAHTA278 | Korczak D; Kister C; Huber B | 2010 | Differentialdiagnostik des Burnout-Syndroms |
| DAHTA279 | Korczak D; Huber B; Steinhauser G; Dietl M | 2010 | Versorgungssituation und Wirksamkeit der ambulanten im Vergleich mit der stationären pneumologischen Rehabilitation |
| DAHTA280 | Schnell-Inderst P; Hunger T; Hintringer K; Schwarzer R; Seifert-Klauss V; Gothe H; Wasem J; Siebert U | 2011 | Individuelle Gesundheitsleistungen |
| DAHTA299 | Buchberger B; Heymann R; Huppertz H; Friepörtner K; Pomorin N; Wasem J | 2011 | Effektivität von Maßnahmen der betrieblichen Gesundheitsförderung (BGF) zum Erhalt der Arbeitsfähigkeit von Pflegepersonal |
| DAHTA300 | Korczak D; Steinhauser G; Dietl M | 2011 | Effektivität von Maßnahmen im Rahmen primärer Prävention am Beispiel kardiovaskulärer Erkrankungen und des metabolischen Syndroms |
| DAHTA301 | Dietl M; Korczak D | 2011 | Versorgungssituation in der Schmerztherapie in Deutschland im internationalen Vergleich hinsichtlich Über-, Unter- oder Fehlversorgung |
| DAHTA309 | Korczak D; Steinhauser G; Dietl M | 2011 | Prävention des Alkoholmissbrauchs von Kindern, Jugendlichen und jungen Erwachsenen |
| DAHTA255 | Balzer K; Bremer M; Schramm S; Lühmann D; Raspe H | 2012 | Sturzprophylaxe bei älteren Menschen in ihrer persönlichen Wohnumgebung |
| DAHTA281 | Hagen A; Gorenoi V; Schönermark MP | 2012 | Knochenersatzmaterialien zur Behandlung von traumatischen Frakturen der Extremitäten |
| DAHTA307 | Aidelsburger P; Schauer S; Grabein K; Wasem J | 2012 | Alternative Methoden zur Behandlung postmenopausaler Beschwerden |
| DAHTA329 | Neusser S; Bitzer EM; Mieth I; Krauth C | 2012 | Medizinische Wirksamkeit und Kosteneffektivität von Minocyclin/Rifampicin-beschichteten zentralvenösen Kathetern zur Prävention von Blutbahninfektionen bei Patienten in intensivmedizinischer Betreuung |
| DAHTA331 | Korczak D; Steinhauser G; Kuczera C | 2012 | Effektivität der ambulanten und stationären geriatrischen Rehabilitation bei Patienten mit der Nebendiagnose Demenz |
| DAHTA332 | Korczak D; Wastian M; Schneider M | 2012 | Therapie des Burnout-Syndroms |

**Table 3: Technology reports with economic assessment based on DAM (n=17); included**

| **Document Number** | **Authors** | **Year** | **Titel** |
| --- | --- | --- | --- |
| DAHTA024 | Aidelsburger P; Hessel F; Wasem J | 2003 | Stellenwert von Ultraschallverfahren im Rahmen der Osteoporoseversorgung (Früherkennung des Frakturrisikos). Ökonomischer Kurz-HTA |
| DAHTA067 | Siebert U; Muth C; Sroczynski G; Velasco-Garrido M; Gerhardus A; Gibis B | 2003 | Dünnschichtpräparationen und computergestützte Untersuchungen von Zervixabstrichen - Medizinische Effektivität, gesundheitsökonomische Evaluation und systematische Entscheidungsanalyse |
| DAHTA069 | Siebert U; Sroczynski G | 2003 | Antivirale Therapie bei Patienten mit chronischer Hepatitis C in Deutschland - medizinische und ökonomische Evaluation der initialen Kombinationstherapie mit Interferon / Peginterferon und Ribavirin |
| DAHTA071 | Corzillius M; Mühlberger N; Sroczynski G; Peeters J; Siebert U; Jäger H; Wasem J | 2003 | Wertigkeit des Einsatzes der genotypischen und phänotypischen HIV-Resistenzbestimmung im Rahmen der Behandlung von HIV-infizierten Patienten |
| DAHTA063 | Kunze S; Schnell-Inderst P; Hessel F; Grill E; Nickisch A; Siebert U; Voß von H; Wasem J | 2004 | Hörscreening für Neugeborene - ein Health Technology Assessment der medizinischen Effektivität und der ökonomischen Effizienz |
| DAHTA110 | Frank W; Konta B; Peters-Engl C | 2004 | Pap-Test zum Screening auf Zervixkarzinom. Einfluss verschiedener Untersuchungsintervalle |
| DAHTA126 | Gorenoi V; Dintsios CM; Hagen A | 2005 | Senkung der Restenoserate durch Einsatz beschichteteter Stents bei koronarer Herzkrankheit. Systematische Übersicht zur medizinischen Wirksamkeit und gesundheitsökonomische Bewertung zum Vergleich von beschichteten gegenüber unbeschichteten Stents |
| DAHTA131 | Zahn J von; Schnell-Inderst P; Gothe H; Häussler B; Menke D; Brüggenjürgen B; Willich S; Wasem J | 2006 | Episiotomie bei der vaginalen Geburt |
| DAHTA137 | Schnell-Inderst P; Kunze S; Hessel F; Grill E; Siebert U; Nickisch A; Voss H von; Wasem J | 2006 | Hörscreening für Neugeborene - Update |
| DAHTA148 | Gorenoi V; Dintsios CM; Schönermark MP; Hagen A | 2006 | Laparoskopische vs. offene Appendektomie - Systematische Übersicht zur medizinischen Wirksamkeit und gesundheitsökonomische Analyse |
| DAHTA193 | Siebert U; Bornschein B; Schnell-Inderst P; Rieber J; Pijls N; Wasem J; Klauss V | 2008 | Messung der fraktionierten Flussreserve zur Indikationsstellung der perkutanen Koronarintervention |
| DAHTA218 | Gorenoi V; Dintsios CM; Schönermark MP; Hagen A | 2008 | Intravaskuläre Brachytherapie bei peripherer arterieller Verschlusskrankheit (PAVK) |
| DAHTA219 | Gorenoi V; Dintsios CM; Schönermark MP; Hagen A | 2008 | Medikamente freisetzende Stents im Vergleich zu Bypass-Operationen bei koronarer Herzkrankheit |
| DAHTA229 | Schmieder K; Engelhardt M; Wawrzyniak S; Börger S; Becker K; Zimolong A | 2010 | Stellenwert der Radiochirurgie von Meningeomen im Vergleich mit der fraktionierten stereotaktischen Bestrahlung, der konventionellen 3D-geplanten konformalen Bestrahlung und der mikrochirurgischen Operation |
| DAHTA265 | Sroczynski G; Schnell-Inderst P; Mühlberger N; Lang K; Aidelsburger P; Wasem J; Mittendorf T; Engel J; Hillemanns P; Petry KU; Krämer A; Siebert U | 2010 | Entscheidungsanalytische Modellierung zur Evaluation der Langzeit-Effektivität und Kosten-Effektivität des Einsatzes der HPV-DNA-Diagnostik im Rahmen der Zervixkarzinomfrüherkennung in Deutschland |
| DAHTA297 | Gorenoi V; Schönermark MP; Hagen A | 2011 | Perkutane Koronarinterventionen zusätzlich zur optimalen medikamentösen Therapie bei stabiler Angina Pectoris |
| DAHTA308 | Gorenoi V; Schönermark MP; Hagen A | 2012 | CT-Koronarangiografie versus konventionelle invasive Koronarangiografie bei der KHK-Diagnostik |

**Table 4: Technology reports without economic assessment (n=31); excluded**

| **Document Number** | **Authors** | **Year** | **Titel** |
| --- | --- | --- | --- |
| DAHTA002 | Lühmann D; Kohlmann T; Raspe H | 1998 | Die Evaluation von Rückenschulprogrammen als medizinische Technologie |
| DAHTA003 | Gibis B; Busse R; Reese E; Richter K; Schwartz FW; Köbberling J | 1998 | Das Mammographie-Screening zur Brustkrebsfrüherkennung |
| DAHTA005 | Pientka L | 1998 | PSA-Screening beim Prostatakarzinom |
| DAHTA006 | Gibis B; Busse R; Schwartz FW | 1999 | Verfahrensbewertung der Magnet-Resonanz-Tomographie (MRT) in der Diagnostik des Mamma-Karzinoms |
| DAHTA007 | Pientka L | 1999 | Minimal-invasive Therapie der benignen Prostatahyperplasie (BPH-Syndrom) |
| DAHTA008 | Röseler S; Duda L; Schwartz FW | 1999 | Evaluation präoperativer Routinediagnostik (Röntgenthorax, EKG, Labor) vor elektiven Eingriffen bei Erwachsenen |
| DAHTA011 | Perleth M; Jakubowski E; Busse R | 1999 | Bewertung von Verfahren zur Diagnostik der akuten Sinusitis maxillaris bei Erwachsenen |
| DAHTA012 | Gernreich C | 1999 | Spezifische Hyposensibilisierung mit Allergenextrakten bei extrinsischem Asthma bronchiale und Insektengiftallergie |
| DAHTA013 | Lühmann D; Kohlmann T; Lange S; Raspe H | 2000 | Die Rolle der Osteodensitometrie im Rahmen der Primär-, Sekundär- und Tertiärprävention/Therapie der Osteoporose |
| DAHTA014 | Röseler S; Schwartz FW | 2000 | Evaluation arthroskopischer Operationen bei akuten und degenerativen Meniskusläsionen |
| DAHTA015 | Fritze J | 2000 | Die Evaluation von Stroke Units als medizinische Technologie |
| DAHTA017 | Perleth M | 2000 | Vergleichende Effektivität und Differentialindikation von Ballondilatation (PTCA) versus Bypasschirurgie bei Ein- und Mehrgefäßerkrankungen der Herzkranzgefäße |
| DAHTA018 | Lühmann D; Hauschild B; Raspe H | 2000 | Hüftgelenkendoprothetik bei Osteoarthrose |
| DAHTA004 | Droste S; Brand A | 2001 | Biochemisches Screening für fetale Chromosomenanomalien und Neuralrohrdefekte - eine Verfahrensbewertung |
| DAHTA022 | Lühmann D | 2001 | Stellenwert der Magnet-Resonanz-Tomographie im Rahmen der Versorgung von Patienten mit Rückenschmerzen - Kurz-HTA: Update einer Best-Evidence-Synthese |
| DAHTA061 | Corzillius M; Pientka L; Siebert U; Wasem J | 2002 | Wertigkeit von Tumor-Nekrose-Faktor alpha-Antagonisten in der Behandlung der rheumatoiden Arthritis (Medizinischer Teil) |
| DAHTA068 | Gorenoi V; Dintsios CM; Perleth M | 2002 | Stenting versus Ballondilatation bei koronarer Herzkrankheit - systematische Übersicht zur medizinischen Effektivität |
| DAHTA010 | Lühmann D; Raspe H | 2003 | Operative Eingriffe an der lumbalen Wirbelsäule bei bandscheibenbedingten Rücken- und Beinschmerzen - eine Verfahrensbewertung |
| DAHTA026 | Dettenkofer M; Merkel H; Mutter J | 2003 | Bewertung unterschiedlicher Hygienekonzepte zur Kontrolle von MRSA (Methicillin-resistente Staphylococcus aureus) |
| DAHTA029 | Schroeder A; Reese E; Richter K; Köbberling J | 2003 | Die Wertigkeit der Streßechokardiographie in der Primärdiagnostik der koronaren Herzkrankheit |
| DAHTA030 | Wild C; Frank W; Konta B; Huber K | 2003 | Medizinische Effektivität beim Einsatz von GP- IIb / IIIa-Rezeptorantagonisten in der Therapie von akuten Koronarsyndromen |
| DAHTA060 | Perleth M; Gerhardus A; Velasco M | 2003 | Positronen-Emissions-Tomographie - systematische Übersichten zur Wirksamkeit bei ausgewählten Indikationen |
| DAHTA065 | Peeters J; Siebert U; Aidelsburger P; Regar E; Rieber J; Wasem J; Klauss V | 2003 | Wertigkeit des Einsatzes der intravaskulären Ultraschallbildgebung (IVUS) im Rahmen von diagnostischen und therapeutischen Herzkatheteruntersuchungen - ein HTA-Bericht zur medizinischen Effektivität |
| DAHTA073 | Mand P | 2003 | Verfahrensbewertung der CT-Angiographie, MR-Angiographie, Doppler-Sonographie und Szintigraphie bei der Diagnose von Nierenarterienstenosen |
| DAHTA078 | Gernreich NC; Gerhardus A; Velasco-Garrido M | 2003 | Knochen- und Knochenersatzmaterialien zur parodontalen Regeneration und zum Knochenaufbau für Implantate - eine systematische Bewertung der medizinischen Wirksamkeit |
| DAHTA072 | Rosery H; Maxion-Bergemann S; Rosery B; Bergemann R | 2004 | Ultraschall in der Schwangerschaft. Beurteilung der routinemäßigen Schwangerschaftsultraschalluntersuchungen unter Maßgabe der Mutterschaftsrichtlinien |
| DAHTA074 | Schroeder A; Heiderhoff M; Köbberling J | 2004 | Stroke Units - Update des HTA Berichts "Die Evaluation von Stroke Units als medizinische Technologie" |
| DAHTA113 | Schroeder A; Heiderhoff M; Köbberling J | 2005 | Bestimmung der Albuminausscheidung im Urin bei Diabetikern zur Vorsorge und Kontrolle der diabetischen Nephropathie |
| DAHTA117 | Lange-Lindberg AM; Velasco-Garrido M; Busse R | 2006 | Misteltherapie als begleitende Behandlung zur Reduktion der Toxizität der Chemotherapie maligner Erkrankungen |
| DAHTA233 | Rasch A; Müller-Riemenschneider F; Vauth C; Willich SN; Greiner W | 2008 | Föderale Strukturen und damit verbundene verhaltensbezogene Maßnahmen zur Prävention des Zigarettenrauchens |
| DAHTA344 | Korczak D | 2012 | Föderale Strukturen der Prävention von Alkoholmissbrauch bei Kindern und Jugendlichen |

**Table 5: Methodological reports (n=20); excluded**

| **Document Number** | **Authors** | **Year** | **Titel** |
| --- | --- | --- | --- |
| DAHTA1 | Bitzer E; Busse R; Dörning H; Duda L; Köbberling J; Kohlmann T; Lühmann D; Pasche S; Perleth M; Raspe H; Reese E; Richter K; Röseler S; Schwartz FW | 1998 | Bestandsaufnahme, Bewertung und Vorbereitung der Implementation einer Datensammlung "Evaluation medizinischer Verfahren und Technologien" in der Bundesrepublik |
| DAHTA9 | Behrend C; Greiner W; Hessel F; Hoffmann C; Leidl R; Mühlberger N; Schulenburg JM Graf von der; Siebert U; Wasem J; Welte R | 1999 | Ansätze und Methoden der ökonomischen Evaluation - eine internationale Perspektive |
| DAHTA25 | Raum E; Perleth M | 2003 | Methoden der Metaanalyse von diagnostischen Genauigkeitsstudien |
| DAHTA27 | Aidelsburger P; Felder S; Siebert U; Wasem J | 2003 | Gesundheitsökonomische "Kurz-HTA-Berichte" - eine systematische Übersichtsarbeit zur Methodik und Implementation |
| DAHTA34 | Ekkernkamp M; Lühmann D; Raspe H | 2003 | Methodenmanual für "HTA-Schnellverfahren" und Exemplarisches "Kurz-HTA": Die Rolle der quantitativen Ultraschallverfahren zur Ermittlung des Risikos für osteoporotische Frakturen |
| DAHTA62 | Droste S; Gerhardus A; Kollek R | 2003 | Methoden zur Erfassung ethischer Aspekte und gesellschaftlicher Wertvorstellungen in Kurz-HTA-Berichten - eine internationale Bestandsaufnahme |
| DAHTA122 | Zentner A; Velasco-Garrido M; Busse R | 2005 | Methoden zur vergleichenden Bewertung pharmazeutischer Produkte |
| DAHTA99 | Siebert U | 2005 | Entscheidungsanalytische Modelle zur Sicherung der Übertragbarkeit internationaler Evidenz von HTA auf den Kontext des deutschen Gesundheitssystems |
| DAHTA31 | Gerhardus A; Dintsios CM | 2006 | Der Einfluss von HTA-Berichten auf die gesundheitspolitische Entscheidungsfindung - eine systematische Übersichtsarbeit |
| DAHTA210 | Neumann U; Hagen A; Schönermark MP | 2007 | Regulation der Aufnahme von innovativen nichtmedikamentösen Technologien in den Leistungskatalog solidarisch finanzierter Kostenträger |
| DAHTA194 | Siebert U; Zietemann V; Sroczynski G | 2008 | Pharmacogenomics-Bias - Systematische Verzerrungen in Studienergebnissen durch genetische Heterogenität |
| DAHTA214 | Kossmann B; Ulle T; Kahl KG; Wasem J; Aidelsburger P | 2008 | Nichtmedikamentöse verhaltensbezogene Adipositastherapie unter Berücksichtigung der zugelassenen Arzneimittelbehandlung |
| DAHTA243 | Schöttker B; Lühmann D; Boulkhemair D; Raspe H | 2009 | Indirekte Vergleiche von Therapieverfahren |
| DAHTA250 | Mangiapane S; Velasco Garrido M | 2009 | Surrogatendpunkte als Parameter der Nutzenbewertung |
| DAHTA251 | Gorenoi V; Schönermark MP; Hagen A | 2009 | Instrumente zur Risikoprädiktion für kardiovaskuläre Erkrankungen |
| DAHTA259 | Gorenoi V; Schönermark MP; Hagen A | 2009 | Gelenkendoprothesenregister für Deutschland |
| DAHTA272 | Bartelmes M; Neumann U; Lühmann D; Schönermark MP; Hagen A | 2009 | Methoden zur frühen entwicklungsbegleitenden Bewertung innovativer medizinischer Technologien |
| DAHTA260 | Dreier M; Borutta B; Stahmeyer J; Krauth C; Walter U | 2010 | Vergleich von Bewertungsinstrumenten für die Studienqualität von Primär- und Sekundärstudien zur Verwendung für HTA-Berichte im deutschsprachigen Raum |
| DAHTA264 | Gorenoi V; Schönermark MP; Hagen A | 2010 | Infektionsschutz in der Knieendoprothetik |
| DAHTA220 | Brettschneider C; Lühmann D; Raspe H | 2011 | Der Stellenwert von Patient-Reported Outcomes (PRO) im Kontext von Health Technology Assessment (HTA) |
